# One health transmission of fluoroquinolone-resistant *Escherichia coli* and risk factors for their excretion by dogs living in urban and nearby rural settings

**DOI:** 10.1016/j.onehlt.2023.100640

**Published:** 2023-10-07

**Authors:** Jordan E. Sealey, Ashley Hammond, Kristen K. Reyher, Matthew B. Avison

**Affiliations:** a University of Bristol School of Cellular & Molecular Medicine, Biomedical Sciences Building, University Walk, Bristol BS8 1TD, United Kingdom; b University of Bristol Medical School, Population Health Sciences, Canynge Hall, 39 Whatley Road, Bristol BS8 2PS, United Kingdom; c University of Bristol Veterinary School, Langford House, Langford, Bristol BS40 5DU, United Kingdom

**Keywords:** Zoonosis, Molecular ecology, Phylogenetics

## Abstract

Rates of fluoroquinolone resistance in *Escherichia coli*, a key opportunistic human pathogen, are problematic. Taking a One Health approach, we investigated the excretion of fluoroquinolone-resistant (FQ-R) *E. coli* by 600 dogs (303 from rural and 297 from urban environments) recruited from a 50 × 50 km region where we have also surveyed FQ-R *E. coli* from cattle and from human urine. FQ-R *E. coli* were detected in faeces from 7.3% (rural) and 11.8% (urban) of dogs. FQ-R *E. coli* from rural dogs tended to be of sequence types (STs) commonly excreted by cattle, whilst those from urban dogs tended to carry plasmid-mediated quinolone resistance genes, common in human *E. coli* in our study region. Phylogenetic evidence was obtained for sharing FQ-R *E. coli* - particularly for STs 10, 162 and 744 - between cattle, dogs and humans. Epidemiological analysis showed a strong association between feeding dogs uncooked meat and the excretion of FQ-R *E. coli*, particularly for STs 10, 162 and 744. This practice, therefore, could serve as a transmission link for FQ-R *E. coli* from farmed animals entering the home so we suggest that dogs fed uncooked meat should be handled and housed using enhanced hygiene practices.

## 1 Introduction

Fluoroquinolones are classed as highest-priority critically important antimicrobials (HP-CIAs) by the World Health Organisation. They are widely used in human and veterinary medicine, including to treat companion and farmed animals [1]. Their bactericidal activity against a broad spectrum of bacterial pathogens, including Gram-negatives, Gram-positives and anaerobes, and their relative safeness, absorption and bioavailability make them a favourable treatment option for such infections [2]. However, their widespread use has driven up fluoroquinolone resistance (FQ-R) rates, and this, in turn, has prompted attempts to reduce fluoroquinolone use in many settings [3].

FQ-R is a global problem, with multiple studies reporting FQ-R in bacteria from humans, animals, and the environment [1,2,4,5]. FQ-R can occur by horizontal transmission of FQ-R genes on plasmids, known as plasmid-mediated quinolone resistance (PMQR) genes [6]. Predominantly, however, resistance is a result of vertical transmission of multiple quinolone resistance-determining region (QRDR) mutations (at least two in *gyrA* and one in *parC*) on the chromosome [7]. Therefore, the movement of bacterial clones harbouring these mutations plays a major role in the transmission of FQ-R. In our recent study of FQ-R *E. coli* from humans (urinary isolates) and dairy cattle (faecal samples) within a 50 km × 50 km study area in the south-west of England, FQ-R was almost always caused by chromosomal mutation [8]. Furthermore, by comparing core genomes of FQ-R *E. coli* from humans and cattle, we showed evidence of general sharing (not though direct transmission) of FQ-R *E. coli* between the two compartments, with as little as 71 (ST744) or 63 (ST162) core genome single nucleotide polymorphisms (SNP) differences being observed [8].

There have been few other studies where core genome comparisons of FQ-R *E. coli* collected within multiple One Health compartments has been attempted. One study compared the genomes of FQ-R *E. coli* pandemic clones ST131 and ST1193 isolated from pet dogs and cats with those collected from other sources, including humans. This study reported evidence of general sharing of between dogs and humans, with 60 core genome SNP differences between ST131 isolates [9].

We have recently reported the molecular ecology of 3rd generation cephalosporin-resistant (3GC-R) *E. coli* excreted by two groups of dogs within our 50 × 50 km study area [10]. One group was recruited in the Mendip district of Somerset, a rural district close to many of the dairy cattle farms previously studied [11]. The other group was recruited in the city of Bristol, a populated urban area, with the two sampling regions centred 32 km apart [10]. We identified evidence of general sharing of 3GC-R *E. coli* between dogs and those found on farms (rural dogs only) or in human urine (urban dogs only) [10]. Epidemiology based on owner-completed questionnaires found that the risks associated with the excretion of 3GC-R *E. coli* by dogs were complex, particularly in urban environments. One clear risk factor found in the rural population was the feeding of uncooked meat [10]. This suggests that feeding uncooked meat to dogs might significantly erode the barriers between One Health compartments, driving the flow of farm-animal origin HP-CIA-resistant *E. coli* into the domestic environment.

Since *E. coli* is the primary causal species for urinary and bloodstream infections in humans in our region, anything that might increase the flow of HP-CIA-resistant *E. coli* into the human population is important. To investigate further, here we report a comparison of FQ-R *E. coli* from 600 dogs, humans, and cattle within our 50 × 50 km study region together with an analysis of behavioural risk factors associated with excretion of FQ-R *E. coli* in dogs.

## 2 Materials and methods

### 2.1 Recruitment

Ethical approval was obtained from the Faculty of Health Research Ethics Committee, University of Bristol (Ref: 89282) alongside Ethical Approval of an Investigation Involving Animals (ref: UB/19/057). Recruitment of 600 adult dogs between September 2019 and September 2020 along with individual faecal sample collection information has been reported previously, alongside age demographics [10]. A standardised questionnaire (Table S1) was provided to collect demographic data and variables chosen as being potentially associated with carriage of ABR *E. coli* in dogs. Faecal samples were collected from dogs into sterile containers immediately after depositing, and were either transported to the laboratory on the day of collection or delivered through the post. Once at the laboratory, samples were refrigerated and processed within 48 h.

### 2.2 Sample processing and selection of FQ-R *E. coli*

A portion of each faecal sample (0.1–0.5 g) was weighed, and PBS was added at 10 mL/g before vortexing and mixing with an equal volume of 50% sterile glycerol. Twenty microlitres of each sample was spread onto Tryptone Bile X-Glucuronide agar plates (Sigma) containing ciprofloxacin (0.5 mg/L) based on EUCAST breakpoints [12] and incubated overnight at 37 °C. The limit of detection for FQ-R *E. coli* using this method was ∼1000 cfu/g of faeces. Putative FQ-R *E. coli* were re-streaked to confirm resistance (taking a maximum of three isolates per sample).

### 2.3 Risk factor analysis

Where variables had multiple choice answers ‘Never, Sometimes, Often, Very Often’ in the dog owner questionnaire, ‘Never’ was collapsed to ‘No’ and all other responses were collapsed to ‘Yes’. Samples from only one dog per household were included in the analysis, with the dog chosen at random prior to data being obtained. Preliminary Chi-squared tests were used to determine associations between the binary variables and sample-level positivity (where one sample represented each dog) for FQ-R *E. coli*. Univariable logistic regression was then performed using all variables to determine crude odds ratios between positivity for FQ-R *E. coli* and each variable in urban and rural dogs separately. Finally, a multivariable logistic regression model was built, one for rural and one for urban dogs, each including all variables where the respective univariable analysis gave a *p* value <0.05. The multivariable models were built using a backward stepwise method and identified statistically significant (*p* < 0.05) variables associated with sample-level positivity for FQ-R *E. coli*. Hosmer-Lemeshow goodness of fit tests were used to test the fit of each final multivariable model.

### 2.4 PCR and WGS

A multiplex PCR assay, was used to identify plasmid-mediated quinolone resistance genes *qnrA*, *qnrB*, *qnrC*, *qnrD*, *qnrS*, *oqxAB, aac(6′)-1b-cr* and *qepA* in FQ-R *E. coli* isolates as previously described [13]. Three additional PCRs were used to identify additional resistance genes in FQ-R isolates: one was specific for *tet*(B) carried on plasmid pMOO-32 [11] and two were multiplex PCRs, one for the five bla_CTX-M_ gene types and one for other common β-lactamase genes *bla*_TEM_, *bla*_OXA-1_, *bla*_SHV_, *bla*_CMY-2_ and *bla*_DHA-1_ [14]. At least one isolate per sample positive for FQ-R *E. coli* was selected for WGS, with multiple isolates from the same sample being sequenced only if they produced different multiplex PCR profiles. WGS was performed by MicrobesNG on a HiSeq 2500 instrument (Illumina, San Diego, CA, USA) using 2 × 250 bp paired end reads. Reads were trimmed using Trimmomatic [15] and assembled into contigs using SPAdes [16] 3.13.0. Contigs were annotated using Prokka [17]. WGS data were analysed using ResFinder 4.1 [18] and STs were designated by MLST 2.0 [19].

### 2.5 Phylogenetics

WGS data from FQ-R canine *E. coli* isolates were compared with data from FQ-R human or dairy farm isolates [8]. WGS data where >500 contigs were present were excluded due to relatively poor assembly. Only one isolate with the same ST and resistance gene profile for each farm, dog or human was used. Sequence alignment and phylogenetic analysis was carried out as described previously [8]; in brief, sequences were aligned using Snippy and Snippy-core, and maximum likelihood trees were generated using RAxML [20] with the GTRGAMMA model of rate of heterogeneity. SNP distances were determined using SNP-dists (https://github.com/tseemann/snp-dists) and phylogenetic trees were illustrated using Microreact (https://microreact.org/) [21]. Relevant reference genomes are shown in Table S2. For statistical comparisons of the prevalence of certain genotypic properties between rural and urban dogs, isolates from only one FQ-R *E. coli*-positive dog per household were included, with the dog chosen at random prior to data being obtained.

## 3 Results

### 3.1 Prevalence and mechanisms of FQ-R in *E. coli* excreted by 600 dogs

Faecal samples were collected from 303 rural dogs (from 274 households) and 297 urban dogs (from 289 households). FQ-R *E. coli* were detected in faecal samples from 7.3% (*n* = 22) and 11.8% (*n* = 35) of rural and urban dogs, respectively. The apparent differences between the positivity rate for FQ-R *E. coli* between the two groups was not statistically significant (χ^2^ *p* = 0.06). Of the 22 rural and 35 urban dogs positive for FQ-R *E. coli,* 61 and 89 isolates, respectively, were analysed using PCRs to detect PMQR genes, common β-lactamase genes and the tetracycline resistance gene *tet*(B). One isolate with a unique multiplex PCR profile per dog was selected for whole genome sequencing (WGS), amounting to a total of 30 rural and 45 urban dog isolates being sequenced (Table S3). On average, there were 1.36 (urban) and 1.32 (rural) representative FQ-R *E. coli* isolates sequenced per FQ-R-positive dog.

WGS analysis of the FQ-R isolates revealed 10 STs and 6 patterns of QRDR mutations among rural dogs and 23 STs and 10 QRDR mutation patterns among urban dogs (Table 1). Ten sequenced FQ-R isolates from urban dogs carried PMQR genes (nine *qnr*, one *aac(6’)Ib-cr*), which was significantly greater than the single PMQR gene-positive FQ-R isolate found among rural dogs (Fisher's exact χ^2^ *p* = 0.04). Of the 11 PMQR gene-positive FQ-R isolates in total, seven (all *qnr*-positive) carried fewer than the minimal three QRDR mutations necessary for FQ-R [7]; these were the only FQ-R isolates with fewer than three QRDR mutations (Table 1). Overall, therefore, FQ-R in *E. coli* isolates from urban dogs was predominantly due to QRDR mutations, accounting for 78% and 97% in urban and rural dogs, respectively.

**Table 1:** STs and QRDR mutation patterns found in FQ-R *E. coli* in rural and urban dogs in south-west UK.

*E. coli ST*		
Rural	Urban	QRDR mutation pattern
ST744 x 15	ST744 x 7, ST744*, ST744^^	*gyrA*(Ser83Leu), *gyrA*(Asp87Asn), *parC*(Ser80Ile), *parC*(Ala56Thr)
ST162 x 5, ST155 x 2, ST93	ST162 x 6, ST533 x 2, ST453, ST1140, ST2973, ST5229	*gyrA*(Ser83Leu), *gyrA*(Asp87Asn), *parC*(Ser80Ile)
ST224 x 2, ST1196	ST2006 x 3, ST1196, ST1196^, ST90, ST90^+^, ST297	*gyrA*(Ser83Leu), *gyrA*(Asp87Asn), *parC*(Ser80Ile), *parE*(Ser458Ala)
ST10, ST1193	ST1193 x 4, ST10	*gyrA*(Ser83Leu), *gyrA*(Asp87Asn), *parC*(Ser80Ile) *parE*(Leu416Phe)
ST131	ST131 x 2	*gyrA*(Ser83Leu), *gyrA*(Asp87Asn), *parC*(Ser80Ile), *parC*(Glu84Val), *parE*(Ile416Leu)
	ST354	*gyrA*(Ser83Leu), *gyrA*(Asp87Asn), *parC*(Ser80Ile), *parC*(Glu84Glu), *parE*(Ile355Thr)
	ST448	*gyrA*(Ser83Leu), *gyrA*(Asp87Asn), *parC*(Ser80Ile), *parE*(Ser458Thr)
	ST212	*gyrA*(Ser83Leu), *gyrA*(Asp87Asn), *parC*(Ser84Lys)
ST7343*	ST1421^, ST4213^^, ST5259^^, ST7366*	*gyrA*(Asp87Asn), *parC*(Ser80Ile)*
	ST88*, ST155*	*gyrA*(Ser83Leu)*

Isolates carry plasmid-mediated quinolone resistance genes ^+^*aac(6’)Ib-cr,* *q*nrS1,* ^q*nrB4, ^^*q*nrB19.*

### 3.2 One Health comparison of FQ-R *E. coli* phylogeny within a 50 × 50 km region

ST744 was the most common ST among sequenced FQ-R isolates from both groups of dogs but was significantly more common in rural dogs (52% of isolates) than in urban dogs (20% of isolates; χ^2^ *p* = 0.004; Table 1). Other STs in common between the two groups included ST10, ST131, ST155, ST1193 and ST1196 and, except for ST155, all shared the same QRDR mutation pattern (Table 1).

We hypothesized that dogs sharing a household would also share *E. coli*. In only 3/28 rural and 1/8 urban multi-dog households sampled did a dog from that household test positive for FQ-R *E. coli* and in only one of these (rural) households did both dogs test positive. WGS analysis confirmed that this pair of dogs was, in fact, positive for FQ-R *E. coli* of different STs.

**Fig. 1:**
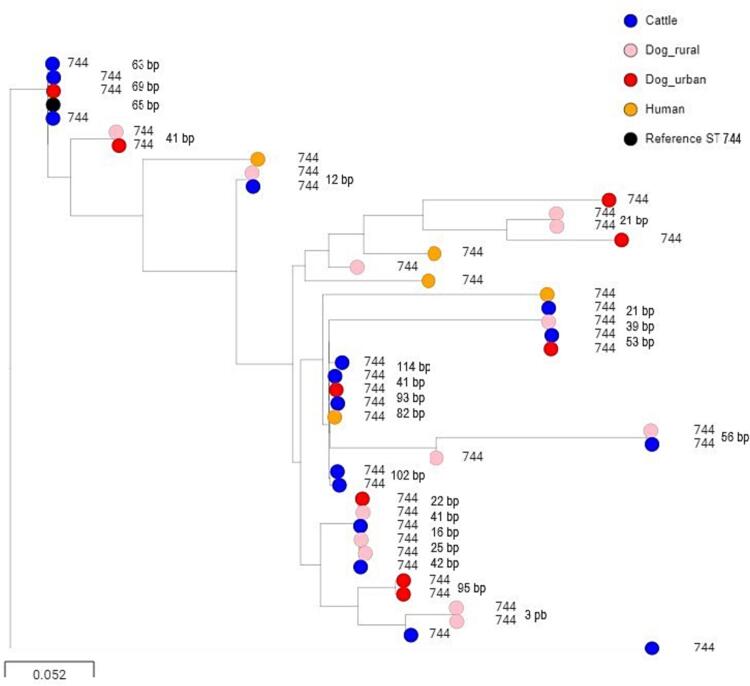
Phylogenetic tree of core genome alignment of FQ-R *E. coli* ST744 isolates from rural and urban dogs, humans and dairy cattle in the south-west region of the UK. SNP distances (bp) are labelled between isolates on the same vertical branch.

Phylogenetic analysis was carried out on 194 FQ-R *E. coli* isolates, including those collected in this study as well as human and dairy cattle isolates reported previously within the same 50 × 50 km study area [8]. A phylogenetic tree was produced based on core genome alignment of all isolates (Fig. S1). Pairs of isolates found in two (or more) One Health compartments appeared closely related for six STs, so ST-specific trees were produced to determine how closely related these isolates were. Figs. 1, S2 and S3 show evidence of sharing (pairwise SNP differences <100, a cut-off that has been previously published [8]) of ST744, ST10, ST162, isolates across all four compartments. Furthermore, ST131 isolates from three dogs were found to be 45, 58 and 92 SNPs different from the closest human isolate. In comparison, the closest pair of human isolates had 33 SNP differences (Fig. S4). For ST1193, minimal SNP distances between isolates from humans and from rural or urban dogs were 51 and 56, respectively (Fig. S5). ST93 isolates were found in one rural dog and two humans only. Detailed analysis identified 32 and 34 SNP differences from a dog isolate and each human isolate, respectively, which was closer than the distance between these two human isolates (44 SNP differences) (Fig. S6).

### 3.3 Risk factor analyses for excreting FQ-R *E. coli* in rural and urban dogs

Preliminary χ^2^ analyses were carried out to determine the significance of associations between variables included in the dog survey (Table S1) and excretion of FQ-R *E. coli* in dogs (Table 2). Univariable analyses using all variables identified the feeding of dry kibble had a negative association with FQ-R *E. coli* excretion in both rural and urban groups (OR 0.19, CI 0.08 to 0.48, *p* < 0.001, and OR 0.37, CI 0.16 to 0.87, p = 0.02, respectively). The feeding of raw (uncooked) meat had a positive association with FQ-R *E. coli* excretion in both rural and urban groups (OR 22.9, CI 95% 8.5 to 62.0, *p* < 0.001, and OR 4.6, CI 2.0 to 10.8, p < 0.001). No significance (*p* ≥ 0.05) was found in the univariable analyses for any other variable and these were excluded from the multivariable logistic regression model, which revealed that feeding raw meat to both rural and urban dogs was associated with increased odds of them excreting FQ-R *E. coli* (OR 18.6, 95% CI 5.8 to 59.8, p < 0.001, and OR 4.0, 95% CI 1.4 to 11.4, *p* = 0.009, respectively. No association was identified between excretion of resistant *E. coli* and walking rural dogs in environments alongside cattle (Table 2). To further investigate this by focusing on dogs that frequently interacted with such environments, we re-evaluated the data by combining categories where the survey question was answered “often/very often” versus “sometimes/never”, but again, no association was identified.

**Table 2:** Chi-squared analyses of potential risk factors associated with excretion of FQ-R *E. coli* in dogs from rural and urban regions. Variables included in the multivariable logistic regression models for rural (*) and urban (†) dogs are highlighted.

		Rural Dogs		Urban Dogs	
Risk factor		Total (*N* = 303)	FQ-R *E. coli* (N = 22)	Total (*N* = 297)	FQ-R *E. coli* (N = 35)
Dry food *†	Yes No ***p*=**	254 49 **≤0.001**	12 10	258 39 **0.019**	26 9
Wet food	Yes No *p*=	122 181 0.489	7 15	121 176 0.054	9 26
Human food	Yes No *p*=	164 139 0.687	11 11	155 142 0.924	18 17
Raw food *†	Yes No ***p*=**	26 277 **≤0.001**	12 10	31 266 **≤0.001**	10 25
Fed raw food in the past	Yes No *p*=	19 284 0.692	0 22	21 276 0.739	2 33
Walking on streets	Yes No *p*=	287 16 0.873	21 1	269 28 0.424	33 2
Walking in parks	Yes No *p*=	288 15 0.928	21 1	294 3 0.258	35 0
Walking on beaches	Yes No *p*=	220 83 0.327	14 8	223 74 0.256	29 6
Walking in the countryside (without livestock)	Yes No *p*=	256 47 0.388	20 2	234 63 0.53	29 6
Walking in the countryside (with livestock)	Yes No *p*=	222 81 0.659	17 5	190 107 0.328	25 10
Walking in countryside (with cattle)	Yes No *p*=	141 162 0.095	14 8	115 182 0.593	15 20
Playing in sea estuary	Yes No *p*=	144 159 0.519	9 13	174 123 0.854	20 15
Playing in lake	Yes No *p*=	81 222 0.953	6 16	106 191 0.575	11 24
Playing in river	Yes No *p*=	165 138 0.65	13 9	162 135 0.293	22 13
Playing in pond	Yes No *p*=	90 213 0.232	9 13	136 161 0.283	19 16
Owning another dog(s)	Yes No *p*=	70 233 0.63	6 16	33 264 0.098	1 34
Owning a cat(s)	Yes No *p*=	42 261 0.059	6 16	51 246 0.63	5 30
Owning rodent(s)	Yes No *p*=	12 291 0.88	1 21	6 291 0.73	0 35
Owning bird(s)	Yes No *p*=	13 290 0.951	1 21	3 294 0.258	0 35
Owning reptile(s)	Yes No ***p*=**	8 295 0.282	0 22	5 292 **0.048**	2 33
Owning horse(s)	Yes No *p*=	7 296 0.467	1 21	0 297 N/A	N/A
Owning livestock	Yes No *p*=	8 295 0.563	1 21	0 297 N/A	N/A
Antibiotic use in last 6 months	Yes No *p*=	44 259 0.613	4 18	42 255 0.29	7 28

We noted sharing of ST10, ST162 and ST744 isolates between cattle and dogs in the phylogenetic analysis (Fig. 1, S2, S3). We hypothesized that this sharing was at least in part due to the feeding of uncooked meat to dogs. After deduplicating isolates by dog and by household, FQ-R *E. coli* isolates from dogs fed uncooked meat were biased (18 versus 9 isolates) towards STs 10, 162 and 744 versus *E. coli* from all other STs. In contrast, FQ-R *E. coli* isolates were biased in the opposite way (13 from STs 10, 162 and 744 versus 22 from all other STs; χ^2^ *p* = 0.02) in dogs not fed uncooked meat.

## 4 Discussion

One of the main aims of this study was to test the hypothesis that within our 50 × 50 km study region, dogs living in rural or urban areas excreted FQ-R *E. coli* related to those found in cattle or humans, respectively. Our study did find some evidence for this; FQ-R *E. coli* from rural dogs had a bias towards ST744 (Table 1), which is common in cattle and rarer in humans in our study region [8]. Urban dogs, however, excreted a wider range of FQ-R *E. coli* STs and had a bias towards carrying PMQR genes (Table 1), which were more common in human than cattle isolates in our study region [8].

It does appear, however, that some FQ-R *E. coli* STs are being widely shared between One Health compartments. ST744, ST162 and ST10 – previously identified in humans and on farms in our study area [8] – were found here in both rural and urban dogs. This fits with other reports of these STs being found in multiple host species, including humans and in the environment [[22], [23], [24]].

Further evidence of sharing FQ-R *E. coli* between humans and dogs (but not cattle) has been reported for clinically important, extra-intestinal pathogenic *E. coli,* ST131 and ST1193 [25,26]. Both STs are frequently associated with FQ-R bloodstream and urinary tract infections in humans, and they were identified in both dog groups in our study (Table 1, Fig. S4, S5), which concurs with studies of companion animals in other countries [26,27]. ST131 and ST1193 are often multi-drug resistant, including CTX-M-mediated 3GC-R [13,[28], [29], [30]]. We did not detect any *bla*_CTX-M_ genes in *E. coli* selected for FQ-R from either dog group in this study, but these genes were detected in other STs in our parallel study of *E. coli* selected for 3GC-R from the same population of dogs [10].

We found that the excretion of FQ-R *E. coli* by both rural and urban dogs was strongly associated with feeding raw meat. This fits with other studies that show an association between raw meat feeding and the excretion of resistant bacteria by dogs [10,[31], [32], [33], [34]]. Notably, we found that excretion of FQ-R *E. coli* of STs 10, 162 and 744, which are common in cattle [8], was particularly associated with feeding raw meat.

In a recent study on puppies (≤16 weeks old), samples from which were collected across the United Kingdom, raw meat feeding was also strongly associated with carriage of FQ-R *E. coli* [34]. The prevalence of FQ-R *E. coli* excretion seen in urban adult dogs in this current study was almost identical to that seen in puppies (11.7% and 11.8% sample-level positivity respectively) where identical sample processing and microbiology methods were employed [34], which fits with a scenario where a national study [34] is dominated by urban animals.

Earlier studies from within our study area found increased FQ use was associated with an increased odds of finding FQ-R *E. coli* on dairy farms [8] and reducing FQ use in humans was associated with reducing FQ-R in primary care-derived urinary *E. coli* [35]. Antimicrobial usage in the dogs in our study was not associated with the odds of those dogs excreting FQ-R *E. coli* (Table 2) and it may take some time for the effects of dramatic FQ usage reduction seen on UK farms (particularly since June 2018) [36] to have an impact on the levels of faecally derived FQ-R *E. coli* contaminating uncooked meat, even if uncooked meat marketed as dog food in the UK is sourced from UK farms, which may not be the case [37].

In conclusion, these analyses of urban and rural canine groups within an extensively studied 50 × 50 km region have provided further evidence of sharing of resistant *E. coli* between dogs, humans and cattle. Excretion of FQ-R *E. coli* was strongly associated with raw meat feeding; more strongly than was excretion of 3GC-R *E. coli* in the same group [10]. This highlights a potentially important route by which FQ-R *E. coli* can enter the home, and so re-emphasises the importance of antimicrobial use reduction on farms from which uncooked meat sold for feeding to dogs comes. Furthermore, it also emphasises the importance of hygienic handling and preparation of uncooked meat (whether for human or companion animal consumption, even if to be cooked) and the requirement for particularly strict hygiene when handling dogs, or faeces excreted by them, if the dogs are fed uncooked meat. Alternatively, dog owners may consider that feeding uncooked meat contaminated with *E. coli*, a bacterium that causes a majority of human urinary tract and bloodstream infections in Western countries [38], resistant to antimicrobials used for the treatment of such infections, poses a risk. Testing of uncooked meat marketed as dog food, and certification that it is free of contamination by such bacteria would likely allay such fears.

## Funding

This work was funded by grant NE/N01961X/1 to M.B.A. and K.K.R. from the Antimicrobial Resistance Cross Council Initiative supported by the seven United Kingdom research councils. J.E.S. was supported by a scholarship from the Medical Research Foundation National PhD Training Programme in Antimicrobial Resistance Research (MRF-145-0004-TPG-AVISO).

## CRediT authorship contribution statement

**Jordan E. Sealey:** Conceptualization, Investigation, Data curation, Project administration, Formal analysis, Writing – original draft, Writing – review & editing. **Ashley Hammond:** Formal analysis, Writing – review & editing. **Kristen K. Reyher:** Conceptualization, Funding acquisition, Supervision, Writing – review & editing. **Matthew B. Avison:** Conceptualization, Funding acquisition, Supervision, Writing – review & editing, Writing – original draft.

## Declaration of Competing Interest

M.B.A. is married to the owner of a veterinary practice that sells various mass-manufactured dog foods amounting to a value <5% of total turnover. Otherwise, the authors declare no competing interests.

## Data Availability

Data will be made available on request.

## Appendix A Supplementary data

Supplementary tables S1-S3 and figures S1-S6 Image 1
