# Supplementary material for: One health transmission of fluoroquinolone-resistant Escherichia coli and risk factors for their excretion by dogs living in urban and nearby rural settings
Source: One Health. 2023 Oct 7;17:100640. doi: 10.1016/j.onehlt.2023.100640 (PMC10665141; doi:10.1016/j.onehlt.2023.100640)

**Supplementary Information for:**

**Molecular ecology and risk factors for fluoroquinolone-resistant *Escherichia coli* carriage by dogs living in urban and nearby rural settings**

**Jordan E. SEALEY^1^, Ashley HAMMOND^2^, Kristen K. REYHER^3^, Matthew B. AVISON^1^***

**^1^University of Bristol School of Cellular & Molecular Medicine, Biomedical Sciences Building, University Walk, Bristol. BS8 1TD. United Kingdom.**

**^2^University of Bristol Medical School, Population Health Sciences, Canynge Hall, 39 Whatley Road, Bristol. BS8 2PS. United Kingdom.**

**^3^University of Bristol Veterinary School, Langford House, Langford, Bristol. BS40 5DU. United Kingdom.**

**Running Title: FQ-R *E. coli* in dogs**

***Corresponding Author: Matthew B. Avison, School of Cellular & Molecular Medicine, University of Bristol. Bristol BS8 1TD. United Kingdom. bimba@bris.ac.uk.**

**Table S1. Dog owner’s questionnaire**

Dog Owner’s Questionnaire

**Investigating the Regional Circulation of Antibiotic Resistance Genes**

Date …………………………………

How old is your dog? …………………………………..

What breed is your dog? ............................................................................................................

1. Which types of food do you regularly feed your dog, or have they been fed in the past? Please tick all that apply.

|  | Currently feeding | Have fed in the past |
| --- | --- | --- |
| Commercial wet food (e.g. tins or pouches) |  |  |
| Commercial dry food (e.g. kibble or pellets) |  |  |
| Commercially prepared raw diet |  |  |
| Home-prepared uncooked/raw meat |  |  |
| Home-prepared cooked meat |  |  |
| Table scraps and leftovers |  |  |
| Any other information: | | |

2. In which of these environments is your dog walked? Please tick all that apply.

|  | Never | Sometimes | Often | Very often |
| --- | --- | --- | --- | --- |
| Roads and streets |  |  |  |  |
| Parks |  |  |  |  |
| Beaches |  |  |  |  |
| Countryside, in spaces shared with livestock animals |  |  |  |  |
| Countryside but not in spaces where livestock animals are found |  |  |  |  |
| In places where cattle are kept |  |  |  |  |

3. Does your dog ever swim, paddle or play in any of the following?

|  | Never | Sometimes | Often | Very often |
| --- | --- | --- | --- | --- |
| Sea or estuary |  |  |  |  |
| Lakes |  |  |  |  |
| Rivers |  |  |  |  |
| Ponds |  |  |  |  |
| Other water source (please describe briefly) | | | | |

4. Do you own any other animals? Please give brief details.

5. Has your dog been unwell and therefore had antibiotics given or prescribed by your vet in the last 6 months? If so, please give brief details of the reason for antibiotic use and what was given.

6. We would be very grateful if you could give us some information about where you regularly walk your dog, particularly in the countryside. For example, you could tell us the road names, park names, map references. postcodes or nearby well-known landmarks.

**Table S2.** Reference genomes used for phylogenetic analysis.

| **ST** | **Accession number** |
| --- | --- |
| ST10 | CP041992.1 |
| ST93 | CP010585.1 |
| ST131 | HG941718.1 |
| ST162 | CP042585.1 |
| ST744 | CP016182.2 |
| ST1193 | CP030111.1 |

**Table S3.** PCR profiles of FQ-R *E. coli* isolated from rural and urban dogs’ faecal samples in the south-west region of the UK. Isolates with the same PCR profiles from the same dog are indicated as ‘(no. of isolates)’.

| Cohort | Dog ID (no. of isolates) | Isolate WGS | Genes amplified in multiplex PCRs |
| --- | --- | --- | --- |
| Rural | 104 (2) | 104-1CIPR | *tet*(B), *bla*_TEM_ |
| Rural | 104 (1) | 104-3CIPR | *tet*(B) |
| Rural | 105 (3) | 105-1CIPR | *tet*(B), *bla*_TEM_ |
| Rural | 129 (1) | 129-1CIPR | *tet*(B) |
| Rural | 133 (2) | 133-1CIPR | *tet*(B), *bla*_TEM_ |
| Rural | 133 (1) | 133-2CIPR | *bla*_TEM_ |
| Rural | 136a (1) | 136a-1CIPR | *tet*(B) |
| Rural | 140 (3) | 140-1CIPR | *tet*(B), *bla*_TEM_ |
| Rural | 165 (3) | 165-1CIPR | *tet*(B), *bla*_TEM_ |
| Rural | 169 (3) | 169-1CIPR | N/A |
| Rural | 188 (3) | 188-1CIPR | *bla*_TEM_ |
| Rural | 235 (3) | 235-1CIPR | *tet*(B), *bla*_TEM_ |
| Rural | 250a (3) | 250a-1CIPR | *tet*(B), *bla*_TEM_ |
| Rural | 269 (3) | 269-1CIPR | *bla*_TEM_ |
| Rural | 270 (2) | 270-1CIPR | N/A |
| Rural | 270 (1) | 270-3CIPR | *tet*(B), *bla*_TEM_ |
| Rural | 271a (3) | 271a-1CIPR | *tet*(B), *bla*_TEM_ |
| Rural | 271b (3) | 271b-1CIPR | N/A |
| Rural | 285 (1) | 285-1CIPR | *bla*_TEM_ |
| Rural | 285 (2) | 285-2CIPR | *tet*(B), *bla*_TEM_ |
| Rural | 286 (2) | 286-1CIPR | *tet*(B), *bla*_TEM_ |
| Rural | 286 (1) | 286-2CIPR | *tet*(B) |
| Rural | 301 (2) | 301-1CIPR | *bla*_TEM_ |
| Rural | 314 (3) | 314-1CIPR | *tet*(B), *bla*_OXA_ |
| Rural | 322 (1) | 322-1CIPR | *bla*_TEM_ |
| Rural | 322 (1) | 322-2CIPR | *tet*(B), *bla*_TEM_ |
| Rural | 322 (1) | 322-3CIPR | *tet*(B), *bla*_TEM_ |
| Rural | 367 (1) | 367-1CIPR | *bla*_TEM_ |
| Rural | 367 (2) | 367-2CIPR | *bla*_TEM_, *qnrS* |
| Rural | 368 (3) | 368-1CIPR | *tet*(B), *bla*_TEM_ |
| Urban | 1011 (3) | 1011-1CIPR | *tet*(B), *bla*_TEM_ |
| Urban | 1030 (1) | 1030-1CIPR | *tet*(B), *bla*_TEM_ |
| Urban | 1050 (3) | 1050-1CIPR | *tet*(B), *bla*_TEM_ |
| Urban | 1058 (1) | 1058-1CIPR | *bla*_TEM_ |
| Urban | 1069 (1) | 1069-1CIPR | *tet*(B) |
| Urban | 1069 (1) | 1069-2CIPR | *bla*_TEM_ |
| Urban | 1069 (1) | 1069-3CIPR | *tet*(B), *bla*_TEM_ |
| Urban | 1070 (3) | 1070-1CIPR | *tet*(B) |
| Urban | 1088 (2) | 1088-1CIPR | *tet*(B), *bla*_TEM_ |
| Urban | 1091 (3) | 1091-1CIPR | *bla*_TEM_ |
| Urban | 1109 (3) | 1109-1CIPR | *bla*_TEM_ |
| Urban | 1110 (1) | 1110-1CIPR | *bla*_TEM_ |
| Urban | 1110 (1) | 1110-2CIPR | N/A |
| Urban | 1110 (1) | 1110-3CIPR | *tet*(B), *bla*_TEM_ |
| Urban | 1112 (1) | 1112-1CIPR | *bla*_TEM_, *qnrS* |
| Urban | 1112 (2) | 1112-2CIPR | *tet*(B), *bla*_TEM_ |
| Urban | 1114 (1) | 1114-1CIPR | *tet*(B), *bla*_OXA_, *bla*_TEM_, *aac(6’)-Ib-cr* |
| Urban | 1115 (3) | 1115-1CIPR | *bla*_TEM_, *qnrS* |
| Urban | 1120 (1) | 1120-1CIPR | *bla*_DHA_, *qnrB* |
| Urban | 1131 (2) | 1131-1CIPR | *qnrB* |
| Urban | 1131 (1) | 1131-3CIPR | *bla*_TEM_ *qnrS* |
| Urban | 1139 (3) | 1139-1CIPR | *bla*_TEM_ |
| Urban | 1149 (1) | 1149-1CIPR | *bla*_TEM_, *qnrB* |
| Urban | 1149 (2) | 1149-2CIPR | *tet*(B), *bla*_TEM_ |
| Urban | 1166 (3) | 1163-1CIPR | *tet*(B), *bla*_TEM_ |
| Urban | 1169 (2) | 1169-1CIPR | *bla*_TEM_ |
| Urban | 1169 (1) | 1169-2CIPR | *bla*_TEM_, *qnrB* |
| Urban | 1171 (3) | 1171-1CIPR | *bla*_TEM_, *qnrB* |
| Urban | 1194 (2) | 1194-1CIPR | *bla*_TEM_ |
| Urban | 1201 (1) | 1201-1CIPR | *bla*_TEM_ |
| Urban | 1203 (3) | 1203-1CIPR | *bla*_TEM_ |
| Urban | 1214b (3) | 1241b-1CIPR | *tet*(B), *bla*_TEM_ |
| Urban | 1217 (3) | 1217-1CIPR | *tet*(B), *bla*_TEM_, *qnrS* |
| Urban | 1221 (3) | 1221-1CIPR | *bla*_TEM_ |
| Urban | 1222 (3) | 1222-1CIPR | *bla*_TEM_ |
| Urban | 1224 (1) | 1224-1CIPR | *tet*(B) |
| Urban | 1225 (1) | 1225-1CIPR | *bla*_TEM_ |
| Urban | 1225 (2) | 1225-2CIPR | N/A |
| Urban | 1229 (3) | 1229-1CIPR | N/A |
| Urban | 1276 (3) | 1276-1CIPR | *bla*_TEM_ |
| Urban | 1277 (1) | 1277-1CIPR | N/A |
| Urban | 1277 (1) | 1277-2CIPR | *bla*_TEM_ |
| Urban | 1279 (2) | 1279-1CIPR | *bla*_TEM_ |
| Urban | 1279 (1) | 1279-2CIPR | *tet*(B) |
| Urban | 1284 (3) | 1284-1CIPR | N/A |
|  |  |  |  |

**Figure S1.** Phylogenetic tree of core genome alignment of FQ-R *E. coli* isolates from rural (pink) and urban (red) dogs, humans (orange) and cattle (blue) from the south-west region of the UK. Isolates labelled with ST.


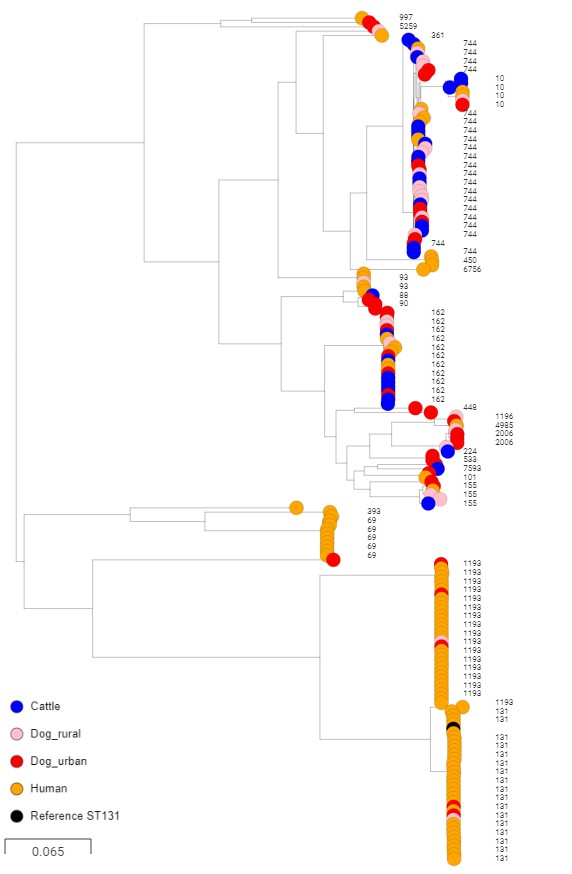


**Figure S2.** Phylogenetic tree of core genome alignment of FQ-R *E. coli* ST10 isolates from a rural and urban dog, a human and cattle in the south-west region of the UK. SNP distances are labelled between isolates on the same vertical branch.

*
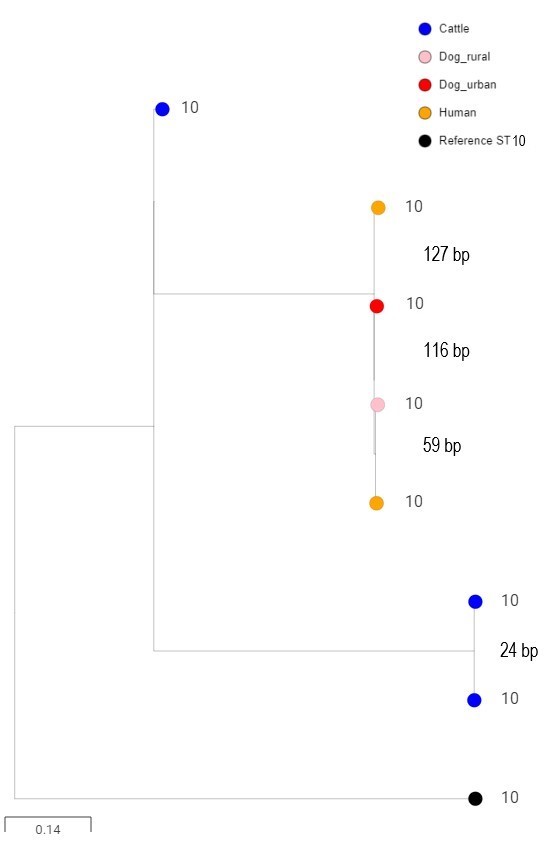
*

**Figure S3.** Phylogenetic tree of core genome alignment of FQ-R *E. coli* ST162 isolates from rural and urban dogs, humans and cattle in the south-west region of the UK. SNP distances are labelled between isolates on the same vertical branch.


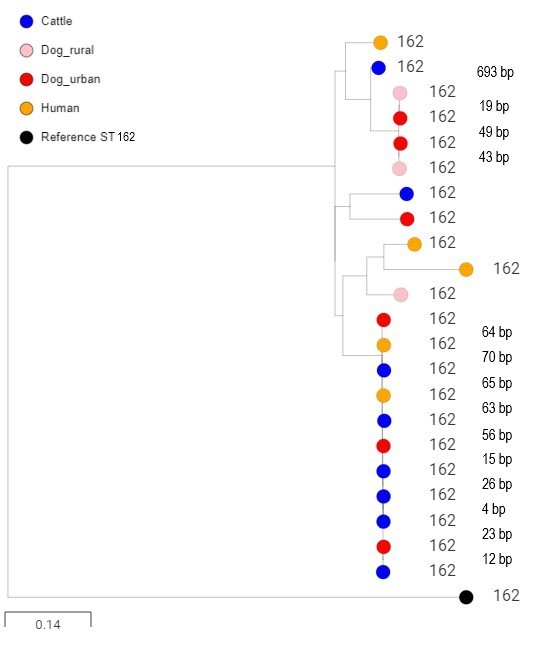


**Figure S4.** Phylogenetic tree of core genome alignment of FQ-R *E. coli* ST131 isolates from a rural dog, urban dogs and humans in the south-west region of the UK. SNP distances are labelled between isolates on the same vertical branch.

*
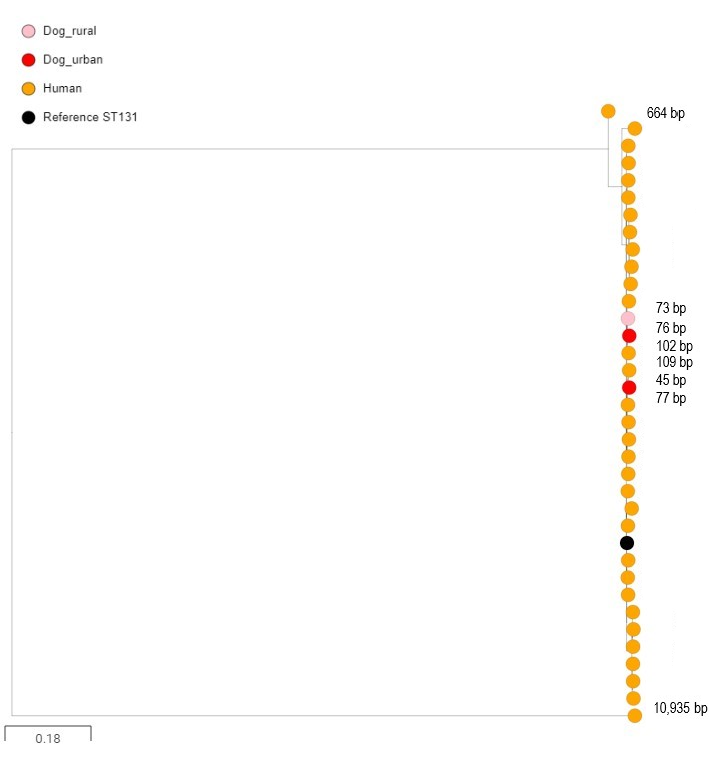
*

**Figure S5.** Phylogenetic tree of core genome alignment of FQ-R *E. coli* ST1193 isolates from a rural and urban dog, and humans in the south-west region of the UK. SNP distances are labelled between isolates on the same vertical branch.


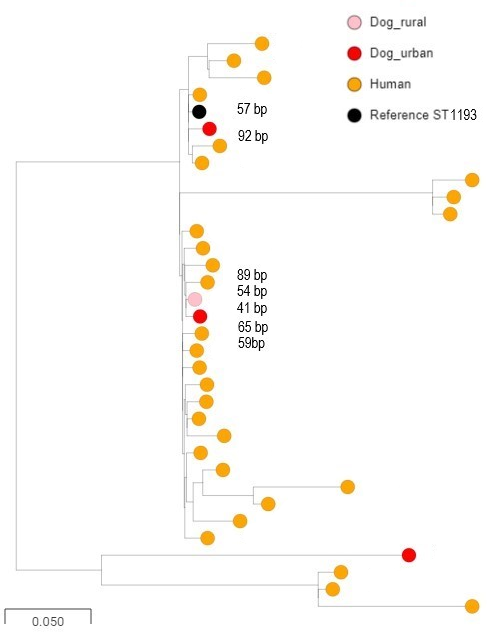


**Figure S6.** Phylogenetic tree of core genome alignment of FQ-R *E. coli* ST93 isolates from a rural dog and humans in the south-west region of the UK. SNP distances labelled between isolates on the same vertical branch.


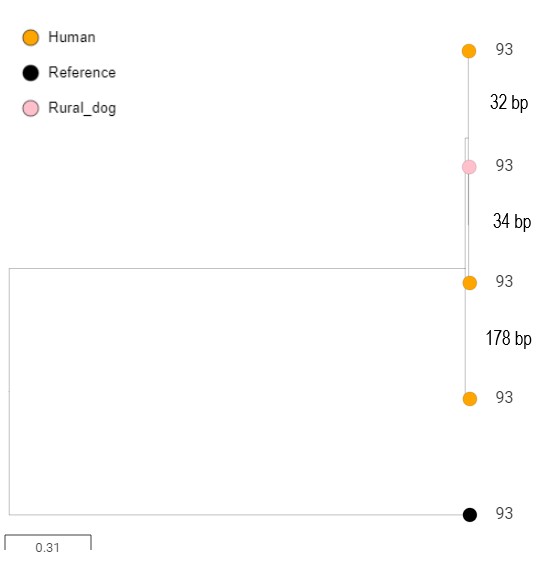

Supplement: Supplementary file 1 — Supplementary tables S1-S3 and figures S1-S6 [file mmc1.docx]
